# Meeting report of the 2016 bone marrow adiposity meeting

**DOI:** 10.1080/21623945.2017.1313374

**Published:** 2017-04-14

**Authors:** Bram van der Eerden, André van Wijnen

**Affiliations:** a Erasmus MC, Department of Internal Medicine, Laboratory for Calcium and Bone Metabolism, Rotterdam, the Netherlands; b Mayo Clinic, Department of Orthopedic Surgery and Biochemistry & Molecular Biology, Rochester, MN, USA

**Keywords:** adipocyte, adiposity, bone marrow, bone marrow adipose tissue, fat

## Abstract

There is considerable interest in the physiology and pathology, as well as the cellular and molecular biology, of bone marrow adipose tissue (BMAT). Because bone marrow adiposity is linked not only to systemic energy metabolism, but also to both bone marrow and musculoskeletal disorders, this biologic compartment has become of major interest to investigators from diverse disciplines. Bone marrow adiposity represents a virtual multi-tissue endocrine organ, which encompasses cells from multiple developmental lineages (e.g., mesenchymal, myeloid, lymphoid) and occupies all the non-osseous and non-cartilaginous space within long bones. A number of research groups are now focusing on bone marrow adiposity to understand a range of clinical afflictions associated with bone marrow disorders and to consider mechanisms-based strategies for future therapies.

## Introduction to the meeting

The topics assembled here were initially discussed at the Second International Meeting on Bone Marrow Adiposity (BMA) in Rotterdam, Netherlands (August 25–26th, 2016) and provide a broad overview of the current state of the field. These meeting notes contain informal conclusions and are intended to encourage the reader to explore them further in literature or by performing own experiments. This meeting was a follow-up of the 1st BMA meeting held in Lille, France.^1^

## Development and characteristics of BMAT

Bone marrow stromal cells (BMSCs) are a heterogeneous group of plastic-adherent cells that include multipotent progenitors. A subset of these cells has the capacity to self-renew and the potential to generate several cell types. BMSCs are related to a much broader group of precursor cells commonly referred to as mesenchymal stem/stromal cells (MSCs), which are ubiquitous fibroblastic cells that can be harvested from many tissues. From a bone perspective, one of the biologically more interesting cell types within a BMSC population is the skeletal stem cell (SSC).^2^ SSCs reside in bone marrow as perivascular cells (pericytes) and represent both skeletal progenitor cells and paracrine regulators of the local bone marrow environment.^2,3^ The ability to generate chondrocytes, osteoblasts, and marrow adipocytes (i.e., multipotency) as well as to recruit haematopoietic progenitors characterizes SSCs The balance of these opposing phenotypes is regulated by extrinsic and intrinsic factors (e.g., growth factors, morphogens), and alterations in these physiologic regulatory mechanisms could lead to skeletal diseases. It has been suggested that excessive accumulation of marrow adipocytes observed in osteoporotic bone loss is caused by disproportional commitment of BMSCs to the adipogenic lineage at the expense of the osteogenic lineage.^4^ For example, in a separation-based anorexia mouse model (SBA) with severe bone loss, BMSCs cultured *in vitro* displayed a dramatic increase in adipogenic differentiation at the expense of osteogenic differentiation.^5^ Another hypothesis for the relationship between increased adiposity and low bone mass postulates that factors (e.g., adipokines) secreted by marrow adipocytes modify the bone marrow microenvironment and may affect the physiologic responses of neighboring cells. This secretome of adipocytes could potentially mediate trans-differentiation of osteoblastic cells toward an adipocyte phenotype as shown in a co-culture model with human primary stem cells.^6^

Whether bone marrow-derived adipocytes share characteristics of peripheral fat cells (white, brown and beige) is still a matter of debate. The adipose organ displays a high degree of plasticity: cold exposure increases the mass and activity of brown adipose tissue (BAT), while an excess in energy enlarges the white adipose tissue (WAT). This observation suggests that adipocytes are prone to convert reversibly under physiologic stimuli to preserve the homeostasis of the organism.^7^ However, in patients with bone marrow transplants, it is evident that precursor adipocytes in human bone marrow contribute to adipogenesis in white subcutaneous fat tissue.^8^ Determining the phenotype of bone marrow-derived adipocytes is crucial with regard to a better understanding of their regulation and function. Bone marrow-derived adipocytes might exhibit both brown and white features. Yet, gene expression profiles of bone marrow-derived versus perigonadal adipocytes from ovariectomized mice are different. This finding indicates phenotypic distinctions between the 2 cell types.^9^ Similarly, comparison of transcriptomes of human bone marrow-derived stromal cells and stromal cells from white adipose tissues revealed differences in the expression of transcription factors and RNA-binding proteins. Moreover, this phenotype varies according to the bone site or bone marrow areas. Electron microscopy observations of human femoral head show the presence of smaller adipocytes interspersed with haematopoietic cells, but larger adipocytes in low-hematopoiesis areas.^10^ In mice, there could be at least 2 distinct populations of bone marrow-derived adipocytes, one that is constitutive and one that is regulated. The “constitutive” type arises early in development and presents a high level of unsaturated fatty acids. “Regulated” bone marrow-derived adipocytes are located in the proximal and mid shaft of the femur and tibia, composed primarily of saturated fatty acids and are sensitive to metabolic and environmental changes.^11^ In conclusion, these data suggest that both developmental and homeostatic regulatory mechanisms may control the abundance of marrow adipocytes and the composition of the bone marrow environment, within the context of bone formation and resorption, as well as systemic energy metabolism.

## Mesenchymal stem cell fate and lineage commitment

The molecular mechanisms controlling stem cell fate and lineage-commitment are fundamental to our understanding of bone marrow adiposity. The biologic properties of the multipotent cells that generate mesenchymal tissues (including bone, cartilage, ligament, tendon, muscle and fat), both during pre- and post-natal development, have remained more elusive than those of pluripotent embryonic stem cells, which are well-defined cells derived from the inner cell mass of blastocysts that can form all somatic tissues and germ-line cells in adult animals. It is well-accepted that the regenerative capacity of these cells declines with age, thus reducing their ability to support musculoskeletal tissue repair. Furthermore, loss of bone in elderly osteoporotic patients frequently occurs concomitantly with increased bone marrow fat. These changes in tissue repair and homeostasis ultimately are associated at the cellular level with mechanisms of self-renewal and cell fate determination in the mesenchymal lineage.

Recent studies indicate that regulation of cell fate in SSCs may be central to maintaining balanced levels of bone marrow adiposity. This premise is based on several key observations made *in vivo* in mouse models with functional mutations that affect either osteogenic or adipogenic lineage differentiation. As mentioned above, at the meeting, several lines of published and unpublished evidence in support of this concept were informally discussed, and are presented here without citable sources of information merely to provide a flavor of the argumentation and the current thinking in the field. First, unscheduled activation of PTH/PTHrP signaling in SSCs suppresses the Wnt inhibitor sclerostin. This suppression results in activation of the osteogenic WNT signaling pathway resulting in excessive bone formation. Second, over-production of erythropoietin by mesenchymal stromal cells (including SSCs), which support hematopoiesis, causes decreased adipocyte formation. Third, mature osteogenic cells exhibit decreased expression of MMP14 (MT-MMP1), which prevents cleavage of a non-canonical ligand for Notch signaling (Delta Like Non-Canonical Notch Ligand 1, DLK1) that is inhibitory for adipogenesis and resides in the extracellular region of osteoblasts. The sequestration of DLK1 on the osteoblast surface precludes its release and the loss of its inhibitory function in adipogenesis contributes to increased bone marrow fat. Fourth, in dyskeratosis congenita,^12^ cells experience accelerated senescence due to pathological shortening of telomeres. This loss of telomere length is linked to progressive bone marrow failure, and decreased haematopoietic marrow occurs concomitantly with increased adipogenic marrow. Taken together, it is becoming increasingly evident that bone marrow adiposity reflects the carefully orchestrated balance of intricate paracrine signals among mesenchymal and haematopoietic stromal cells that control lineage allocation into osteogenic or adipogenic cell fates.

Consistent with the importance of paracrine signaling, studies by Clabaut and colleagues suggest that adipocytes originating from MSCs may promote trans-differentiation (or re-programming) of osteogenic cells derived from MSCs. This trans-differentiation is presumably due to activation of OCT4 (POU5F1) and PPARϒ2 (PPARG), as well as epigenetic changes in CpG methylation due to as-yet undefined adipokines secreted by adipocytes. Studies by van Eerden and colleagues suggest that osteogenic differentiation of MSCs may involve a positive feed-back loop featuring auto/paracrine production of leptin. Hence, osteogenic vs. adipogenic lineage commitment may be the net result of multiple competing growth factors and morphogens that interact with the cell surface of uncommitted and/or pre-committed mesenchymal cells.

Beyond cell-surface-mediated events that involve complex ligand/receptor interactions, MSCs from different sources exhibit major differences in the expression of nuclear proteins that control transcription of mRNAs and their subsequent processing. Unpublished RNA-seq data obtained for blood-vessel-associated pericytes from peripheral adipose-tissue vs. bone marrow revealed hundreds of differentially expressed genes, and several tens of transcription factors and RNA binding proteins that are distinct between the 2 MSC types. These studies showed evidence of novel DNA- and RNA-binding proteins that are selectively upregulated in adipose-derived MSCs, and that are necessary for adipogenic but not osteogenic differentiation. These findings suggest that MSCs from adipose-tissue selectively activate key regulatory proteins for adipogenic differentiation, presumably to prime these cells for repair or expansion of the tissue in which they are embedded.

Collectively, recent studies in the field indicate that clinical perturbations in bone marrow adiposity affect paracrine signaling among mesenchymal and non-mesenchymal cells at different levels of lineage maturation. These paracrine events clearly drive self-renewal and cell fate of mesenchymal cells, and may favor normal lineage progression (programming) or trans-differentiation (re-programming) by altering epigenetic events, as well as expression of both DNA- and RNA-binding gene regulatory factors.

## Endocrine and metabolic regulation of BMAT

BMAT increases with aging and in diverse clinical conditions such as osteoporosis, anorexia nervosa and caloric restriction, Cushing's syndrome, estrogen deficiency, glucocorticoid therapy, and perhaps in obesity and diabetes.^13,14^ The association of these conditions with altered endocrine and metabolic functions raises 2 key questions. First, how do endocrine and metabolic changes impact BMAT formation? Second, does BMAT itself exert endocrine and/or metabolic effects similar to those observed for WAT or BAT?

Several reports presented at *BMA2016* provided new insights to these questions. Aging has an important influence on bone structure and function, and is a factor with a sex-dependent component related to temporal changes in gonadal hormone production. Osteoporosis is a significant health problem for which postmenopausal women are most vulnerable. Gina Woods reported her group's analysis of the AGES study (Age, Gene/Environment Susceptibility) of older subjects (∼78–79 y old), which revealed that free estradiol negatively correlates with vertebral BMAT in males but not females. This is consistent with the concept that estrogens suppress BMAT formation (at least in elderly men) and indicates a potential mechanism for the sex-dependent effects of estrogen. Also, treatment of postmenopausal women with 17β-estradiol decreases BMAT within 2 weeks, independent of changes in bone mass.^15^ Thus, while no association was found between endogenous estradiol and BMAT in postmenopausal women, it is clear that exogenous estradiol in such subjects can potently and rapidly suppress BMAT.

In addition to aging and osteoporosis, there is much interest in the relationship between BMAT and obesity. Indeed, obesity is defined by excessive adiposity and is associated with metabolic and endocrine dysregulation^16^ as well as adverse effects on skeletal health.^17^ In a mouse obesity model, BMAT increases after 2 weeks of high-fat diet feeding,^18^ which is similar to findings recently published elsewhere.^19^ However, it was noted that BMAT is not consistently altered during obesity in humans. In agreement with this observation, obesity status does not alter multi-lineage differentiation potential (‘stemness’) or proliferation capacity of human BMSCs. Nevertheless, another human study found that obesity enhances the chemoattractant properties of bone marrow-derived adipocytes, which indicates that obesity can influence at least some BMAT characteristics in humans.

The obesity pandemic has motivated a surge of research into BAT, which, via uncoupling protein 1 (UCP1), can mediate adaptive thermogenesis and thereby enhance energy expenditure. As such, there is now much interest in the relationship between ambient temperature, adiposity, and metabolic function.^20^ In an elegant study it was shown that housing growing mice at thermoneutrality increases cancellous bone and BMAT.^21^ This highlights a new consideration for the relationship between BMAT, metabolic homeostasis, and skeletal health. Related to this, previous studies have detected *Ucp1* transcripts in whole bones, leading to speculation that BMAT has BAT-like characteristics.^22^ However, it was reported that, even following treatment with thiazolidinediones (TZDs), expression of UCP1 protein is undetectable in whole tibiae. This argues against the concept that BMAT is BAT-like, despite some evidence that long-term cold exposure can stimulate UCP1 expression in BMAT of rodents.

Several reports addressed the function of BMAT during caloric restriction (CR). In the mouse SBA model of anorexia, it was found that there is a significant positive correlation between BMAT volume and circulating adiponectin. Conversely, CR in rabbits does not increase BMAT or circulating adiponectin,^23^ suggesting that BMAT accumulation is required for CR-induced hyperadiponectinaemia. Thus, these observations support previous findings that BMAT contributes to increased circulating adiponectin during CR.^24^ This endocrine function of BMAT might also occur during treatment with thiazolidinediones (TZDs), in which increased circulating adiponectin is closely associated with adiponectin expression in tibial BMAT, but not in subcutaneous WAT.^25^ Studies by Cawthorn *et al.* in rabbits and rats further revealed that CR decreases leptin expression but increases adiponectin expression in tibial BMAT, suggesting that CR directly alters the endocrine properties of BMAT.

The endocrine causes of BMAT accumulation during CR also remain unresolved. Olfa Ghali reported that SBA mice have increased BMAT, and their BMSCs have elevated expression of adipocyte genes (e.g., Hydroxysteroid 11-β Dehydrogenase 1; 11 β-HSD1 or Hsd11b1) and increased acetylation of Runx2 and Foxo1; these effects also occur in BMSCs following Sirt1 inhibition. Thus, SBA suppresses Sirt1, resulting in increased acetylation of Runx2 and Foxo1, and possibly stimulation of local glucocorticoid activity. Consistent with this finding, CR-induced increases in BMAT are associated with glucocorticoid excess but can occur without hypoleptinaemia.^23^ Thus, there are several potential mechanisms that link CR to increased BMAT. Whether this is also true for BMAT formation in other contexts is unclear. For example, Cawthorn and coworkers reported that mice lacking 11 β-HSD1 were found to have a normal BMAT phenotype, contrary to results of previous studies.^26^ These findings argue against local glucocorticoid action as a key regulator of BMAT development under normal physiologic conditions. Moreover, central leptin administration acts in a β3-AR-dependent manner to robustly decrease ‘regulated’ BMAT, underscoring the concept that leptin can potently modulate BMAT. However, the impact of endogenous leptin on BMAT formation, during CR and in other contexts, remains to be firmly established.

The above studies have focused on CR in non-obese animal models. One recent paper shows that, in obese mice, weight loss limits BMAT accumulation.^19^ However, very little is known about the effect of CR on BMAT in obese humans and how this compares to the well-established responses of serum lipids and other adipose depots. Dimitrios Karampinos and his group reported MRI-based BMAT analyses, including a 2015 study of obese women undergoing CR for 4-weeks.^27^ These analyses showed that BMAT positively correlates with serum cholesterol, LDL, and LDL/HDL before caloric restriction, corroborating previous studies.^28^ However, post-CR there was no correlation between BMAT and serum lipids. CR decreased liver fat, visceral WAT, subcutaneous WAT and circulating lipids, but did not consistently change BMAT: in some subjects BMAT decreased while in others it increased. These changes correlated positively with subcateneous (sc) WAT volume (i.e., BMAT tended to decrease in patients with lower scWAT volume and increase in patients with higher scWAT volume). These findings show that BMAT responds differently to CR than do other adipose depots, which is partially consistent with the finding that, in lean animals and humans, CR increases BMAT.

Finally, Beate Lanske studied a mouse model with conditional deletion of the PTH receptor (*Pth1r*) in MSCs. These mice had substantially increased BMAT, high *Rankl/Tnfsf11* expression in BMAT, and increased Rankl protein in serum and bone marrow; however, no Rankl was detected in peripheral adipose depots. This suggests that BMAT can influence circulating Rankl concentrations, highlighting a novel potential endocrine function of BMAT.^29^

## BMAT and hematopoiesis

In 1882, Neumann noted the inverse relationship between adipogenesis and hematopoiesis within the human bone marrow and documented the predominance of yellow adipocytic marrow in distal bones and the infiltration of distal sites by red haematopoietic marrow in disorders of inefficient or malignant hematopoiesis. It is now well known that red-to-yellow marrow transitions occur in all scenarios of haematopoietic insult, whether due to haematopoietic toxicity or haematopoietic stem cell (HSC) insufficiency. However, the direct role of BMAT on the regulation of HSCs and the subsequent production of the different haematopoietic lineages within the bone marrow is still ill defined. Naveiras and colleagues determined a net negative effect of bone marrow-derived adipocytes in hematopoiesis, both in homeostasis and upon stress-induced hematopoiesis following irradiation-mediated aplasia. More specifically, their work demonstrated that highly adipocytic areas within the marrow were significantly depleted in short-term HSCs and progenitors, as were HSCs co-cultured with fully differentiated adipocytes. Of note, long-term HSC activity remained unchanged after co-culture with either preadipocytes or fully mature adipocytes. Pharmacological or genetically-engineered adipocytic inhibition upon radiation-induced aplasia significantly accelerated haematopoietic recovery after HSC transplantation while enhancing new bone deposition. Others have since validated both the reduced supportive capacity of bone marrow derived mature adipocytes^30^ and the potent effect of PPARG inhibitors in accelerating haematopoietic recovery.^31-33^

In the particular case of aplastic anemia, characterized by massive adipocytic infiltration upon autoimmune HSC insult, a direct effect of PPARG inhibitors in T cell function has also been demonstrated.^32^

Mechanistically, how these findings integrate the recent understanding of the HSC niche is still an open question. The inherent 3D structure of the haematopoietic stem cell (HSC) niche embedded within the bone marrow, hardly accessible for *in vivo* imaging or histological reconstruction at the single cell level, has led to much controversy regarding the nature of the cellular components that directly associate with haematopoietic stem cells (HSCs), constituting the so-called HSC niche. HSCs were first functionally and geographically associated to osteoblasts and the endostium. It has since been determined that although osteoblasts can produce trophic factors that favor HSC maintenance indirectly via the PTH receptor (Pth1r), calcium–sensing mechanisms, N-cadherin interactions and osteopontin, it is mainly Leptin receptor postive perivascular cells (Lepr+) within the highly vascularized endostium that are functionally required for HSC survival through secretion of high levels of CXCL12 for retention and SCF, angiopoietins as well as IGF-like proteins as HSC trophic factors. In parallel, distinct perivascular nestin^GFP^ cells with mesenchymal stem cell (MSC) properties have also been shown necessary and sufficient for HSC self-renewal *in vivo*.^34^ Both perivascular Lepr+ stromal cells and nestin^GFP^ MSCs overlap with the histologically distinct CXCL12-high adventicial reticular cells (CARs) known to associate with HSCs within the bone marrow.^35,36^ In fact, Lepr+ perivascular stromal cells were shown to give rise to most bone marrow adipocytes in the adult bone marrow.^33^ Moreover, it was determined that pre-adipocytes, which are positive for Pref1 (synonym: Delta Like Non-Canonical Notch Ligand 1, DLK1), support hematopoiesis and exert a strong regulatory effect on immune cells that may impact engraftment and bone marrow repopulation by protecting tissues from insult of alloreactive cells. It was shown that cultured cells from digested bone chips contain a heterogeneous population of mesenchymal stem/progenitor cells and preadipocytes and these cells are proficient in adipocytic differentiation. In long-term cultures, human bone marrow adipocytes can support the survival of haematopoietic stem cells.^10^

The Naveiras group also presented an *in vitro* system to mimic the yellow-to-red marrow transition to uncover novel promoters of the yellow-to-red bone marrow transition. Using Digital Holographic Microscopy, they screened the Prestwick Chemical Library of FDA-approved drugs and natural compounds for inhibitors of adipocytic differentiation to accelerate the yellow–to-red marrow transition to improve post-transplant survival. Also they developed and optimized a semi-automated image analysis (MarrowQuant ImageJ Plug-In) that allows for unbiased quantification and size distribution of bone marrow adipocytes *in vivo* from stained bone marrow sections. These approaches have great potential for improving our understanding of the relationship between BMAT and hematopoiesis.

Collectively, the data discussed in the meeting is compatible with the scenario that mature adipocytes, and thus BMAT, prevent the rapid expansion of short-term haematopoietic progenitors while supporting the survival of the most primitive HSC compartment. Contrarily, the preadipocytic Lepr+, CXCL12+ adventitial reticular cells would be responsible for HSC proliferation. Further research is needed to elucidate the specific mechanisms responsible for the differential regulation of adult hematopoiesis throughout the adipocytic differentiation axis, and to determine how deregulation of the adipocytic axis may contribute to the microenvironment-guided initiation or progression of haematopoietic malignancies, as previously demonstrated for osteoblastic and neural-derived signaling within the bone marrow.^37-39^

## BMAT and cancer

It has long been known that interactions between cancer cells and cells of the host bone marrow microenvironment are essential to drive both cancer-induced bone disease and tumor growth and survival. The contributions of osteoblasts and osteoclasts to this relationship are well documented but the role of bone marrow-derived adipocytes remains largely unexplored. Since both the incidence of bone metastases and the proportion of bone marrow-derived adipocytes increase with age, it is tempting to speculate that bone marrow-derived adipocytes may play an important role in supporting tumor cell survival within bone. Murine models have demonstrated that increasing bone marrow adiposity promotes the progression of osteolytic prostate cancer growth within bone.^40^ *In vitro* co-culture systems have revealed a range of potential mechanisms, including lipid transfer from adipocytes to prostate cancer cells, which promotes growth and invasion of the metastasizing cancer cells. Combining a murine model of prostate cancer bone metastases with a high-fat diet revealed a change in tumor cell metabolism toward the Warburg phenotype, with increased glycolytic enzymes, increased lactate production and decreased oxidative phosphorylation. This finding was supported by *in silico* analysis of patients with metastatic prostate cancer, where an increase in genes associated with the Warburg effect was detected.^41^ In addition to metabolic changes, bone marrow adipocytes are emerging as previously overlooked major sources of chemokines and adipokines. In prostate cancer, periprostatic adipocytes have recently been found to play a major role in disease progression via secretion of CCL7.^42^ This same mechanism, using the CCR3/CCL7 axis, has also been found to be adopted by bone marrow-derived adipocytes and to promote migration and homing of bone metastatic prostate cancer cells. Notably, because blockade of this axis prevents metastasis, this axis may represent a potential therapeutic target. In addition to those solid tumors that frequently metastasize to bone, hematological malignancies, including multiple myeloma and acute myeloid leukemia (AML), are also likely to be heavily influenced by bone marrow-derived adipocytes. It has previously been shown that increased adiposity promotes myeloma pathogenesis.^43^ *In vivo* studies using a murine model of myeloma have now identified distinct changes in bone marrow-derived adipocytes within the myeloma-bone microenvironment. For example, *in vitro* co-cultures of myeloma cells and bone marrow-derived adipocytes show that adipocytes promote myeloma cell growth and survival. Similarly, bone marrow-derived adipocytes were found to transfer lipid to leukemic blasts and thereby promote AML proliferation, a biologic effect that uses novel mechanisms mediated by the FABP4 transporter protein in bone marrow-derived adipocytes, and CPT1 in leukemic blasts. It is becoming increasingly evident that bone marrow-derived adipocytes are ideally placed to interact with tumor cells and contribute to the tumor-bone microenvironment. As such, elucidating the relationship between these cell types represents a new avenue to explore novel therapeutic targets for the treatment of these deadly malignancies.

## BMAT and skeletal health

Several recent studies have focused on identifying the bone marrow adipocyte-osteoblast progenitor cell *in vivo*. Although young C57BL/6 mice are largely devoid of BM adipocytes, a small number of perilipin(*Plin1*)-positive:*osterix (Osx1/Sp7)* traced cells were observed in the bone marrow of 8-week-old mice.^44^ Osterix was thought to be expressed solely in osteoblasts, but the lineage-tracing results have raised the possibility of a bi-potent progenitor capable of generating osteogenic and adipogenic cells within the bone marrow. In a separate set of experiments, the majority of CFU-Fs (94%) were found to be traced by expression of the leptin receptor (Lepr).^45^ Lepr also traced most adipocytes and osteoblasts in adult bone marrow. Notably, others have reported that a Gremlin 1 (GREM1)-expressing cell in the bone marrow can self-renew and give rise to osteoblasts, chondrocytes and reticular marrow stromal cells, but not adipocytes.^46^ Based on these data, it appears that bone marrow adipocytes are derived from bone marrow resident mesenchymal progenitor cells. It remains to be established whether this is a single population of progenitors or a small number of different progenitors.

In several conditions, including aging and osteoporosis, the tendency of MSCs to differentiate toward osteoblasts switches in favor of adipogenesis. Of interest, mice held at temperatures below 26°C (optimum is 32°C) gain bone marrow fat and lose bone mass, further supporting the interrelationship between adipogenesis and osteogenesis.^21^ Given the close proximity of BMAT to bone tissue, one of the principal questions is whether bone marrow adipocytes affect skeletal health by biologic effects on their neighboring bone cells including osteoblasts, osteocytes and osteoclasts.

Evidence is accumulating that lipids in the bone marrow microenvironment affect bone cell function. For example, storage of BMAT-derived lipids in osteocytes may lead to their apoptosis, either during osteocyte differentiation or when osteocytes are fully mature and embedded in the bone matrix. Furthermore, saturated free fatty acids (FFA), such as palmitate, cause endoplasmic reticulum stress and apoptosis in human MSCs. Interestingly, Dalla Valle showed that the deleterious effect of saturated FFA was abrogated by converting them into mono-unsaturated FFAs through increased activity of the enzyme stearoyl-Co-A desaturase 1 (SCD1), for example by expression of the Liver X receptor (LXR). This result was supported by a study from the same group looking at patients having non-traumatic osteonecrosis. Among other potential causes, increased bone marrow adiposity may underlie osteonecrosis and it was shown that MSCs from these patients are more susceptible to palmitate, leading to increased adipocyte differentiation. The mechanism proposed to limit the exposure to saturated FFAs as described by Dalla Valle was recapitulated in this study, as shown by dysregulation of ERK (MAPK1/MPAK3) activation and Carnitine Palmitoyltransferase (*CPT1*) and *SCD1* expression, hence leading to lipotoxicity in MSCs from osteonecrotic patients. In line with these preclinical findings are population-based data by Woods and others, who determined bone marrow adiposity of elderly men and women with BMD measurements by magnetic resonance and assessment of the occurrence of prevalent vertebral fractures. Both in men and women, the bone marrow fat percentage positively correlates with prevalent vertebral fracture and femoral neck BMD. Taking an opposite approach, Grahnemo and colleagues studied a RID transgenic mouse model, which is characterized by low visceral adiposity and possibly also low marrow fat. Compared to wild-type mice, both male and female RID mice had increased trabecular BMD and cortical content, as well as greater bone strength. Nevertheless, it is currently unclear whether the improved skeletal health in these mice is explained by low visceral fat or perhaps reduced BMAT. Although many questions remain, these studies together suggest that BMAT, through secretion of FFAs, may affect neighboring bone cells leading to compromised skeletal health.

Ovariectomy in mice leads to rapid bone loss and increased BMAT. By scrutinizing the gene content of BMAT in comparison to peripheral fat tissue, Lucas and colleagues found high expression of MMPs, RANK ligand and 3 Wnt inhibitors (i.e., sFRP4, sFRP1 and Dkk1) and the expression of these genes was further elevated following ovariectomy. Of interest, Bisschop showed that BMAT in women varies with specific stages of pregnancy, which further corroborates the importance of the gonadal axis in regulating BMAT.

Douni presented 2 different transgenic mouse models of osteoporosis. One model expresses low copy numbers of RANKL and presents with trabecular bone loss only; in contrast, the second model has a high RANKL copy number and displays trabecular bone loss and cortical bone porosity, at least partially due to high osteoclast numbers. Of interest, BMAT is increased up to 100% in these high-copy number mice by only 4 months of age.

The Lanske group presented data indicating a novel role for PTH in marrow adipogenesis. Of interest, PTH levels are high in conditions that are characterized by high BMAT, such as osteoporosis, caloric restriction and chronic kidney disease, suggesting that PTH might stimulate adipogenesis. Contrary to this expectation, mice lacking the PTH/PTHrP receptor (Pth1r) in MSCs had severe loss of trabecular and cortical bone with a concomitant marked increase in BMAT. Culturing bone marrow derived cells from these mice exhibited preferential differentiation into adipocytes. Remarkably, bone resorption was also increased in these mice presumably due to BMAT-derived *Rankl* expression. PTH treatment *in vivo* inhibited BM adipogenesis. Hence, this study suggests that PTH signaling may mediate mesenchymal stem cell fate allocation in the bone marrow environment and that BMAT controls bone resorption.^29^

Using an elegant 3D BMAT culture model composed of silk scaffolds, Fairfield showed that another classical osteocytic factor, Sclerostin (SOST), affects BMAT. Leptin has a well-established function as being an appetite-reducing adipokine, but its effect on bone metabolism remains obscure, despite many efforts focusing on the contribution of systemic and peripheral leptin. Van der Eerden and coworkers added complexity to the current knowledge by providing additional evidence that human osteoblasts produce leptin, and that osteoblast-produced leptin is crucial for osteoblastic differentiation and mineralization. It remains unclear whether leptin produced by neighboring adipocytes contributes to osteogenic differentiation, but it is evident that an autocrine/paracrine role in osteoblasts cannot be ignored. Because PTH, sclerostin and leptin collectively represent crucial players in skeletal homeostasis, it will be informative to examine their roles in the potential cross talk between adipocytes and osteoblast lineage cells.

In summary, evidence is accruing that BMAT is a complex tissue in which multiple cell types from distinct developmental lineages control skeletal health through local production of lipids and growth factors. It will be important to determine the temporal and spatial mechanisms that mediate cross-talk of bone cells with BMAT, and how this cross-talk supports skeletal health.

## Future perspective

BMAT research is an emerging and exciting topic in pre-clinical and clinical research. It has direct implications for many different scientific fields, including bone biology, oncology, metabolism, aging, endocrinology, haematology, and rheumatology, but also for BMAT-related clinical conditions. The future research on BMAT will yield novel fundamental knowledge with huge potential for translation, including how BMAT impacts disease progression and by identifying BMAT biomarkers for improved disease diagnosis and treatment. Such knowledge could open new avenues for personalized medicine, as well as population-level screening and management.

## Appendix A. Contributors

• **Peter Arner**. Department of Medicine, Karolinska Institute, Stockholm, Sweden
• **Peter Bisschop**. Academic Medical Center and Free University of Amsterdam, Amsterdam, The Netherlands
• **William Cawthorn, Karla Suchaki, Richard Sulston**. The University of Edinburgh, Edinburgh, United Kingdom
• **Christophe Chauveau, Odile Broux, Pierre Hardouin, Aline Clabaut, Olfa Ghali Mhenni, Stephanie Lucas**. ULCO University, Boulogne sur mer, France
• **Saverio Cinti, Antonella Poloni, Domenico Mattiucci**. Politecnica Delle Marche, University of Ancona, Italy
• **Eleni Douni**. Agricultural University of Athens and Biomedical Sciences research Center “Alexander Fleming,” Athens, Greece
• **Claire Edwards, Emma Morris**. University of Oxford, Oxford, United Kingdom
• **Céline Gillet**. Universite Libre De Bruxelles, Brussels, Belgium
• **Adrien Guérard**. IPBS - CNRS UMR5089, Toulouse, France
• **Louise Grahnemo**. University of Gothenburg, Göteborg, Sweden
• **Mark Horowitz**. Yale University, New Haven, CT, USA
• **Karin Jöhrer**. Tyrolean Cancer Research Institute, Innsbruck, Austria
• **Dimitrios Karampinos**. Technical University of Munich, Munich, Germany
• **Beate Lanske**. Harvard School of Dental Medicine, Boston, USA
• **Olaia Naveiras, Vasco Campos, Shanti Rojas-Sutterlin, Josefine Tratwal**. Ecole Polytechnique Fédérale de Lausanne (EPFL), Lausanne, Switzerland
• **Guillaume Penel**. University of Lille, Lille, France
• **Izabela Podgorski, Jonathan Diedrich**. Wayne State University School Of Medicine, Detroit, USA
• **Jeanine Prompers**. Eindhoven University of Technology and UMC Utrecht, Eindhoven, The Netherlands
• **Mara Riminucci**. Universita degli Studi di Roma ‘La Sapienza’, Rome, Italy
• **Pamela Robey**. National Institute of Dental and Craniofacial Research, Bethesda, USA
• **Clifford Rosen, Heather Fairfield**. Maine Medical Center Research Institute, Scarborough, USA
• **Erica Scheller**. Washington University in Saint Louis, Saint Louis, USA
• **Manar Shafat**. University of East Anglia, Norwich, United Kingdom
• **Michaela Tencerova**. University of Southern Denmark, Odense, Denmark
• **Bram van der Eerden, Patric Delhanty**. Erasmus MC, Rotterdam, The Netherlands
• **André van Wijnen**. Mayo Clinic, Rochester, USA
• **Gina Woods**. University of California, San Diego, La Jolla, USA
